# Transcranial Doppler Ultrasonography detection on cerebrovascular flow for evaluating neonatal hypoxic-ischemic encephalopathy modeling

**DOI:** 10.3389/fnins.2023.962001

**Published:** 2023-05-12

**Authors:** Jin-Xiang Liu, Chang-Le Fang, Kun Zhang, Rui-Fang Ma, Hong-Su Zhou, Li Chen, Qiu-Lin Wang, Yu-Xuan Lu, Ting-Hua Wang, Liu-Lin Xiong

**Affiliations:** ^1^Department of Anesthesiology, Affiliated Hospital of Zunyi Medical University, Zunyi, Guizhou, China; ^2^Laboratory Animal Department, Kunming Medical University, Kunming, China; ^3^School of Anesthesiology, Southwest Medical University, Luzhou, China; ^4^Shantou Ultrasonic Instrument Research Institute Co., Ltd., Shantou, Guangdong, China; ^5^Institute of Neurological Disease, Translational Neuroscience Center, West China Hospital, Sichuan University, Chengdu, China; ^6^Department of Clinical Medicine, Chongqing Medical University, Chongqing, China; ^7^Yunnan Key Laboratory of Primate Biomedical Research, Kunming University of Science and Technology, Kunming, China

**Keywords:** Transcranial Doppler Ultrasonography, neonatal hypoxic-ischemic encephalopathy modeling, cerebrovascular flow, cerebral blood vessels, cerebral infarct

## Abstract

**Objective:**

This study aimed to investigate the feasibility of Transcranial Doppler Ultrasonography (TCD) in evaluating neonatal hypoxic-ischemic encephalopathy (NHIE) modeling through monitoring the alteration of cerebrovascular flow in neonatal hypoxic-ischemic (HI) rats.

**Methods:**

Postnatal 7-day-old Sprague Dawley (SD) rats were divided into the control group, HI group, and hypoxia (H) group. TCD was applied to assess the changes of cerebral blood vessels, cerebrovascular flow velocity, and heart rate (HR) in sagittal and coronal sections at 1, 2, 3, and 7 days after the operation. For accuracy, cerebral infarct of rats was examined by 2,3,5-Triphenyl tetrazolium chloride (TTC) staining and Nissl staining to simultaneously verify the establishment of NHIE modeling.

**Results:**

Coronal and sagittal TCD scans revealed obvious alteration of cerebrovascular flow in main cerebral vessels. Obvious cerebrovascular back-flow was observed in anterior cerebral artery (ACA), basilar artery (BA), middle cerebral artery (MCA) of HI rats, along with accelerated cerebrovascular flows in the left internal carotid artery (ICA-L) and BA, decreased flows in right internal carotid artery (ICA-R) relative to those in the H and control groups. The alterations of cerebral blood flows in neonatal HI rats indicated successful ligation of right common carotid artery. Besides, TTC staining further validated the cerebral infarct was indeed caused due to ligation-induced insufficient blood supply. Damage to nervous tissues was also revealed by Nissl staining.

**Conclusion:**

Cerebral blood flow assessment by TCD in neonatal HI rats contributed to cerebrovascular abnormalities observed in a real-time and non-invasive way. The present study elicits the potentials to utilize TCD as an effective means for monitoring the progression of injury as well as NHIE modeling. The abnormal appearance of cerebral blood flow is also beneficial to the early warning and effective detection in clinical practice.

## Introduction

Neonatal hypoxic-ischemic encephalopathy (NHIE) is most commonly caused by insufficient supply of blood and oxygen to the brains of perinatal newborns (Kyu et al., 2016; Huang et al., 2017), 10% of which are confronted with death, and 30% surviving infants endure severe neurodevelopmental disorders including cognitive impairment, hearing problems, epilepsy, intellectual disability, cerebral palsy, etc (Shankaran et al., 2005; Selway, 2010; Jacobs et al., 2013). The occurrence of NHIE usually can be attributed to fetal distress in uterus caused by umbilical cord around the neck and abnormal amniotic fluid, asphyxia and hypoxia during delivery and after birth (Natarajan et al., 2018; Packer et al., 2022). In normal conditions, adequate cerebral blood flow delivers oxygen and glucose to the fetal brain, helping the fetal brain maintain homeostasis and meet cellular energy demands. However, sudden hypoxia and ischemia disrupts the delivery of oxygen and glucose in the umbilical cord as a result of a decrease in cardiac output and reduction of cerebral blood flow (Smyser et al., 2019). The traced cerebral vascular system is composed of six main arteries including anterior cerebral artery (ACA), middle cerebral artery (MCA), posterior cerebral artery (PCA), and anterior choroidal artery (Ach) originated from internal carotid artery (ICA) and the basilar artery (BA), superior cerebellar artery originated from the vertebral artery (Courties et al., 2015). Many brain pathologies can be diagnosed by visualized alterations of vascular structures and pathology. For instance, ischemic stroke is usually triggered by severe vasospasm in the vascular territories of MCA and ACA (Gubskiy et al., 2018). Cerebral blood flow has been viewed as a helpful biomarker in many cerebrovascular disorders (Carr et al., 2021). It’s been increasingly important to monitor the cerebrovascular abnormality in the related diseases. Transcranial Doppler also plays a potential role in the early detection of cognitive-affective disorders. For example, insufficient cerebral perfusion and increased vascular resistance suggest a correlation with vascular cognitive impairment-dementia-free (VCI-ND) (Vinciguerra et al., 2019). TCD is an effective tool for early detection, evaluation and management of VD patients at risk of dementia (Puglisi et al., 2018).

Inadequate blood flow into corresponding brain regions result in insufficient delivery of oxygen and glucose, which further induces tissues damage and loss. Major rat cerebral arteries closely resemble their human counterparts with regard to the structure of the vascular wall and morphological changes associated with cerebral vascular diseases (Lee, 1995). Rats have made great contributions into mounting explorations of pathogenesis and pathophysiology of NHIE, along with countless achievements of therapeutic targets and treatments for NHIE. Hereof, it is a key step to establish a satisfying NHIE model in the beginning of investigations. Most previous studies verified the induction of NHIE in rats using behavioral tests and morphological detection. However, a clear observation of the cerebrovascular blood flow in rats effectively determines whether the NHIE model is successfully established (Kyng et al., 2015; Lyu et al., 2021).

**FIGURE 1:**
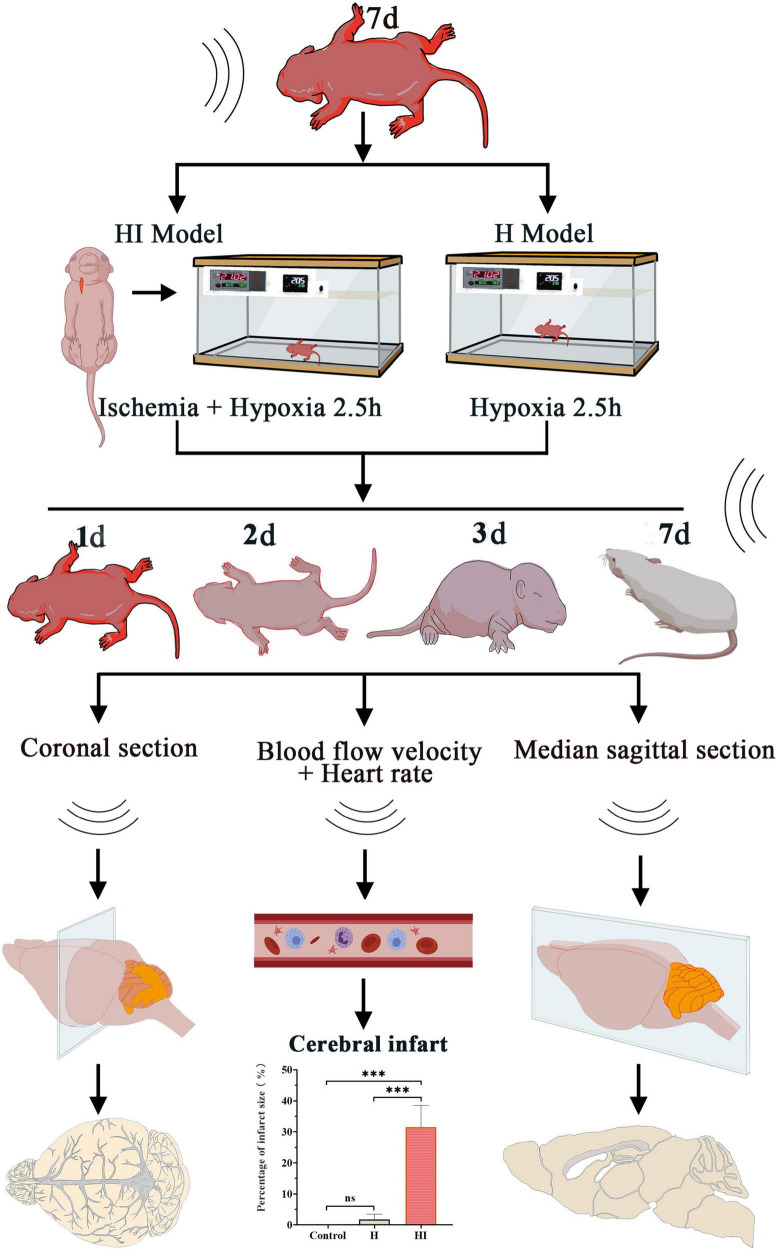
Graphic experimental procedures in this study.

Transcranial Doppler Ultrasonography (TCD) is a rapid, non-invasive imaging method of observing cerebral blood vessels (Lin et al., 2018), which plays an crucial role in the diagnosis of cerebrovascular diseases, such as subarachnoid hemorrhage, vasospasm, and brain death (Guan et al., 2017). A study by Guadagni et al. (2020) found that aerobic exercise modulated cerebrovascular flow and improves cognitive function in the elderly by using TCD to test cerebral blood flow velocity. Although TCD has been used to stratify the severity of ischemic brain damage, studies have seldom reported the application of TCD in validating the success establishment of NHIE model by detecting cerebral arterial hemodynamics. Therefore, this study observed the cerebral vascular structures and cerebral vascular flow noninvasively in NHIE rats under TCD, through which the alteration and progression of HI-induced damage can be dynamically monitored in the absence of animal sacrifice (Figure 1). This study also determined the applicable efficacy of TCD in identifying the successful establishment of the NHIE model.

This study explores the cerebrovascular changes of neonatal rats after HI insult under TCD detection. At 1, 2, 3, and 7 days after HI and H modeling, the cerebral vessels and blood flow rate were detected in the coronal and sagittal positions, and the heart rate was measured according to the average heart rate of the left and right internal carotid artery. The infarct size was detected by TTC staining and the Nissl staining of whole brain was performed in control group, H group and HI group.

## Materials and methods

### Animals and grouping

Specific pathogen free (SPF) neonatal SD rats (7-day-old) were provided by Department of Experimental Animals, Kunming Medical University and divided into three groups, including the control group, hypoxia-ischemia (HI) group, and hypoxia (H) group (*n* = 7/group). Several vulnerable rats died in the experiments, and there were seven rats remained in control group, five rats in H group and five rats in HI group for later experiments. Animals were housed in the cages with their mothers at a temperature (21–25°C), humidity (45–50%) and light-controlled room. All procedures on neonatal rats were approved by the Ethics Committee of Kunming Medical University (approval number: kmmu20220748), and the rats were cared in compliance with Guide for Care and Use of Laboratory animals in Declaration of Helsinki.

### Establishment of HI and H rat models

Isoflurane (Shandong Ante Animal Husbandry Technology Co., Ltd., Shandong, China) inhalation was used until the neonatal rats were deeply anesthetized. The neonatal rats were fixed supinely, disinfected with iodophor applied to midplane of the neck. The skin of the neck was incised with surgical scissors, then separated layer by layer with forceps to expose the right common carotid artery. After the right common carotid artery separated with micro forceps, the vessel was coagulated by employing monopolar microsurgical electrocoagulation (Spring Medical Beauty instrumentality Co., Ltd, Wuhan, China). Subsequently, the subcutaneous tissue and skin were sutured and finally disinfected with topical iodophor. The animals were put back into their cages for 1 h after regaining consciousness, and fed with 100–300 μl tap water (18–23°C) with a 1 ml syringe. Then, they were placed into an anoxic chamber (Hangzhou Aipu Instrument Equipment Co., Ltd., Hangzhou, China) in which a gas mixture containing 6.8–7.4 % O_2_ and 93.2–92.6 % N_2_ at a flow rate of 1 L/min was maintained continuously for 2.5 h with temperature controlled at 34 ± 1°C. Rats in the HI group suffered both ischemia and hypoxia procedures, while rats in the H group suffered hypoxia treatment with common carotid artery exposure. Control rats were treated with common carotid artery exposure only.

### TCD scanning of neonatal rats and data acquisition

The changes of cerebral hemodynamics in rats were detected using TCD (Apogee 5300, Shantou Ultrasonic Instrument Research Institute Co., Ltd., Shantou, China) (Luo et al., 2022). TCD detected cerebral vessels, coronary blood flow velocities and heart rate (HR) in SD rats at 1, 2, 3, and 7 days after modeling in sagittal and coronal sections. Color flow (CF) is used for the study of the index presenting cerebrovascular flow. When the CF mode was applied, an indicator bar will appear on the left or right side of the screen. The upper end of the indicator bar displays the color toward the blood flow, and the lower end displays the color away from the blood flow, and the blood flow toward the probe was indicated in red and the blood flow away from the probe was indicated in blue. The animals were placed in the prone position with the probe perpendicular to the head to obtain images from the coronal and sagittal positions. L-type linear array probe was selected and applied with coupling agent. In detail, the probe mark points turn toward the right side of the animal in a horizontal (coronal) direction scanning or toward the top of head in a vertical (sagittal) direction scanning, with the screen mark points were in the default position (the left side of the screen). The ultrasound images will be projected on the left side of the screen, and the images were optimized by constantly adjustment of gain, depth and focal length during the entire operation. HR was measured by averaging the HR of the right and left internal carotid arteries.

### Tissue harvest

After TCD detection finished at 7th day, rats were anesthetized with 3% isoflurane (inhalation anesthesia). For 2,3,5-Triphenyl tetrazolium chloride (TTC) staining, once deep anesthesia was achieved, the brains were quickly removed and brain tissues were harvested to be stored in −20°C refrigerator. For Nissl staining, the anesthetized rats were perfused with 0.9 % normal saline (CISEN, Jining, China) followed by fixation with 4% paraformaldehyde (Biosharp, Shanghai, China). Then they were killed and the harvested brains were put into 4% paraformaldehyde for more than 72 h. With paraffin embedded, the brain sections (5 μm) were prepared for Nissl staining.

### TTC staining

Cerebral infarction was measured by TTC staining. Once deep anesthesia was achieved with 3% isoflurane, then brains were quickly removed and transferred into the −20°C refrigerator for 20 min. After freezing, the brains were cut into five coronal slices (2 mm each), and then incubated with 2% TTC solution (Sigma, St. Louis, MO, USA) at 37 °C for 5 min. After dyeing, fixation with 4% polyformaldehyde was followed for 24 h and section images were captured on the next day.

### Nissl staining

Paraffin-embedded tissues being sectioned and deparaffinized, were then stained with 30 μl of 1% cresyl violet solution (Solarbio, Beijing, China) in a wet incubator for 9 min. After washes with distilled water, Nissl Differentiation solution was added onto the sections for 2 min. Subsequently, 95% ethanol was added for swift differentiation until Nissl bodies were purple and other tissues were colorless. Finally, specimens were dehydrated by absolute ethyl alcohol (Tianjin Youpu Chemical Reagent Co., Ltd., Tianjin, China), transparentized by xylene (Solarbio, Shanghai, China) and sealed by neutral gum (Biosharp, Shanghai, China). Images of the brain tissues were captured using a light microscope (Leica, Solms, Germany).

### Zea-longa scores

The Zea-longa scoring was used to assess the neurological deficits of rats. The Zea-longa scoring correlates with the severity of the neurological deficits. Briefly, the neurobehavioral scores of the rats were evaluated at 2 days after HI. The scoring criteria were as follows: (1) 0 point (no neurological deficits - normal behavior); (2) 1 point (mild neurological deficits - the left forelimb can’t be fully extended); (3) 2 points (moderate neurological deficits - rats cannot go straight and walk forward, and the body continues to turn to one side); (4) 3 points (severe neurological deficit - rats are unable to stand, felling to the left when standing); (5) 4 points (loss of consciousness).

### Statistical analysis

All data were imported into SPSS 22.0 for statistical analysis. Data of cerebral blood flow velocities in H group or HI group at different timepoints were analyzed by one-way repeated measures analysis of variance (ANOVA) and independent sample *T* test. Cerebral infarction data among three groups were compared using one-way ANOVA. Data meeting the normal distribution were analyzed by one-way ANOVA with Bonferroni analysis for data under equal variance condition or Dunnett’s analysis for data under uneven variance condition. Kruskal-Wallis test was used for multi-group comparisons that do not conform to normal homogeneity and variance homogeneity. All data were expressed as mean ± standard deviation (SD). *P* values less than 0.05 indicated statistically significant difference, and represented by *p < 0.05, ^**^p < 0.01, ^**^p < 0.001.

## Results

### Alteration of cerebrovascular flows of neonatal HI rats

**FIGURE 2:**
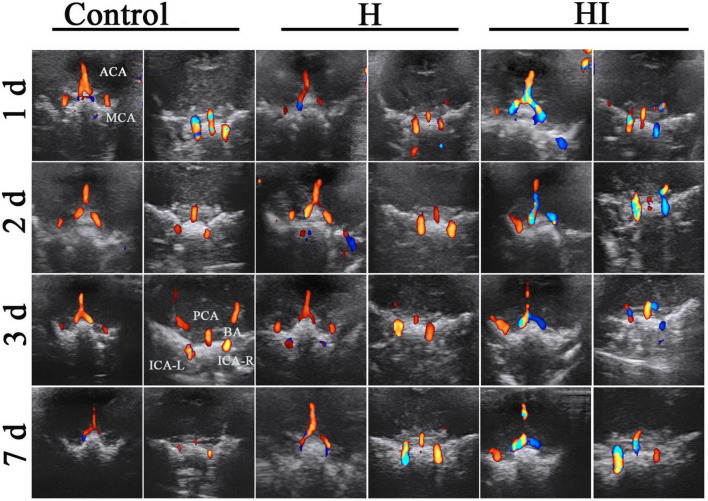
Coronal dynamic acquisition of ultrasound detection of cerebral vessels. Cerebral vessels flows observation of rats in the control (*n* = 7), H (*n* = 5), and HI groups (*n* = 5) at 1, 2, 3, and 7 days after the operations in the coronal position. ACA, anterior cerebral artery; MCA, middle cerebral artery; PCA, posterior cerebral artery; BA, basilar artery; ICA-L, left internal carotid artery; ICAL-R, right internal carotid artery; H, hypoxia; HI, hypoxia and ischemia; d, days.

**FIGURE 3:**
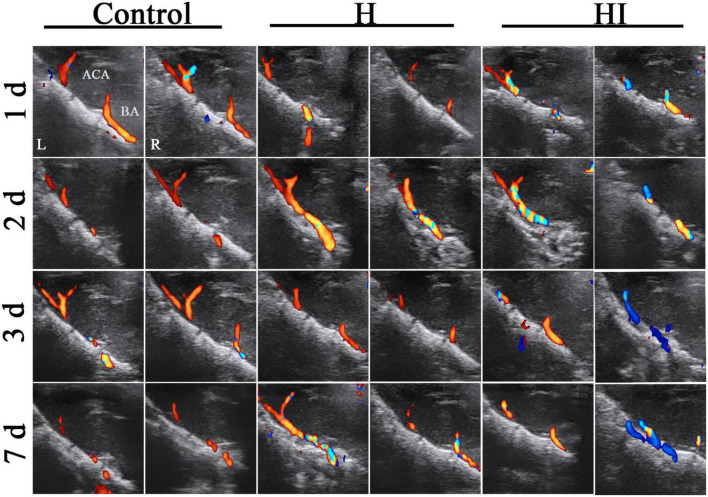
Sagittal dynamic acquisition of ultrasound detection of cerebral vessels. Left and right cerebral vessels in the sagittal position observed at 1, 2, 3, and 7 days after operations in the control (*n* = 7), H (*n* = 5), and HI (*n* = 5) groups. ACA, anterior cerebral artery; BA, basilar artery; H, hypoxia; HI, hypoxia and ischemia; d, days; L, left; R, right.

The cerebral vascular flows in ACA, MCA, PCA, BA, right internal carotid artery (ICA-R), and left internal carotid artery (ICA-L) were dynamically observed under TCD at 1, 2, 3, and 7 days after injury. There was no significant difference of rats in the H group and the control group, indicating that the cerebral blood flows in cerebral vascular system were maintained with appropriate blood supply to cerebral parenchyma in control and H rats. Comparatively, the cerebral blood flows in the ACA, MCA, and ICA-L of HI rats were increased and regurgitated since the right common carotid artery was ligated (in blue) (Figure 2). Similarly in sagittal section, the cerebral blood flows in ACA and BA of control rats and H rats didn’t exhibit any obvious changes within 7 days (Figure 3), while the flows in right ACA and BA were elevated and regurgitated (in blue) at 3 and 7 days after HI injury (Figure 3).

### Dynamic cerebrovascular flow velocity in H and HI rats

**FIGURE 4:**
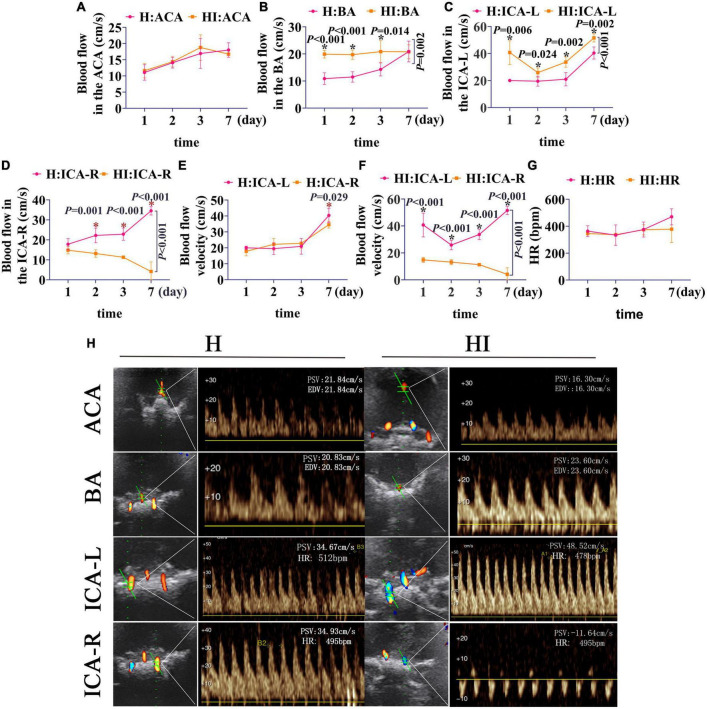
Coronal dynamic detection of cerebrovascular flow velocity and HR in rats. Comparison of flow velocities of the **(A)** ACA, **(B)** BA, **(C)** ICA-L, and **(D)** ICA-R between rats in the H (*n* = 5) and HI (*n* = 5) groups. **(E)** The flow velocities of the ICA-L and ICA-R in the H group. **(F)** The flow velocities of the ICA-L and ICA-R in the HI group. **(G)** Alteration of HR between the H and HI groups. **(H)** Detection of cerebrovascular flow velocities and HR in each vessel under TCD. ACA, anterior cerebral artery; BA, basilar artery; ICA-L, left internal carotid artery; ICA-R, right internal carotid artery; H, hypoxia; HI, hypoxia and ischemia.

To further identify the particular applicable value of TCD on NHIE injury instead of hypoxia injury, we compared the cerebrovascular blow velocities in ACA, BA, ICA-L, and ICA-R of HI and H rats (Figure 4H). No significant differences in the flow velocities of the ACA between the H and HI groups were found at the different time points (Figure 4A), whereas the flow velocities in the BA, ICA-L, and ICA-R in the H and HI groups were significantly different at 1 day (*p* < 0.001 for BA, *p* = 0.006 for ICA-L), 2 days (*p* < 0.001 for BA, *p* = 0.024 for ICA-L, *p* = 0.001 for ICA-R), 3 days (*p* = 0.014 for BA, *p* = 0.002 for ICA-L, *p* < 0.001 for ICA-R), 7 days (*p* = 0.002 for ICA-L, *p* < 0.001 for ICA-R) after injury (Figures 4B–D and Table 1). Flow velocities of BA and ICA-L in the HI group were significantly higher than those in the H group (*p* = 0.002 for BA, *p* < 0.001 for ICA-L), while the opposite was true in ICA-R (*p* < 0.001) flow velocities (Figures 4B–D). In H rats, there was no prominent difference in the flow velocities in ICA-R and ICA-L (Figures 4E, H and Table 1), while the flow velocities in the ICA-R were significantly lower than that in ICA-L in the HI rats (Figures 4F, H and Table 1, *p* < 0.001) at 1 day (*p* < 0.001), 2 days (*p* < 0.001), 3 days (*p* < 0.001), and 7 days (*p* < 0.001) after injury. Moreover, HR of H and HI rats demonstrated no difference at different time points (Figure 4G).

**TABLE 1:** A detailed statistical results of Figure 4.

Dependent variables	Groups	Method of detection	*P*-value
Blood flow in the BA	H; HI	Two-way Repeated Measures ANOVA	0.002
Blood flow in the BA	H; HI (1 days)	*T* Test	<0.001
Blood flow in the BA	H; HI (2 days)	*T* Test	<0.001
Blood flow in the BA	H; HI (3 days)	*T* Test	0.014
Blood flow in the ICA-L	H; HI	Two-way Repeated Measures ANOVA	<0.001
Blood flow in the ICA-L	H; HI (1 days)	*T* Test	0.006
Blood flow in the ICA-L	H; HI (2 days)	*T* Test	0.024
Blood flow in the ICA-L	H; HI (3 days)	*T* Test	0.002
Blood flow in the ICA-L	H; HI (7 days)	*T* Test	0.002
Blood flow in the ICA-R	H; HI	Two-way Repeated Measures ANOVA	<0.001
Blood flow in the ICA-R	H; HI (2 days)	*T* Test	0.001
Blood flow in the ICA-R	H; HI (3 days)	*T* Test	<0.001
Blood flow in the ICA-R	H; HI (7 days)	*T* Test	<0.001
Blood flow in the ICA-R	H (2 days; 7 days)	One-Way ANOVA	0.032
Blood flow velocity in the H group	ICA-R; ICA-L (7 days)	*T* Test	0.029
Blood flow velocity in the HI group	ICA-R; ICA-L	Two-way Repeated Measures ANOVA	<0.001
Blood flow velocity in the HI group	ICA-R; ICA-L (1 day)	*T* Test	<0.001
Blood flow velocity in the HI group	ICA-R; ICA-L (2 days)	*T* Test	< 0.001
Blood flow velocity in the HI group	ICA-R; ICA-L (3 days)	*T* Test	<0.001
Blood flow velocity in the HI group	ICA-R; ICA-L (7 days)	*T* Test	<0.001

ACA, anterior cerebral artery; BA, basilar artery; ICA-L, left internal carotid artery; ICA-R, right internal carotid artery; HR, heart rate; H, hypoxia; HI, hypoxia and ischemia.

### Morphological verification of HI-induced cerebral damage

**FIGURE 5:**
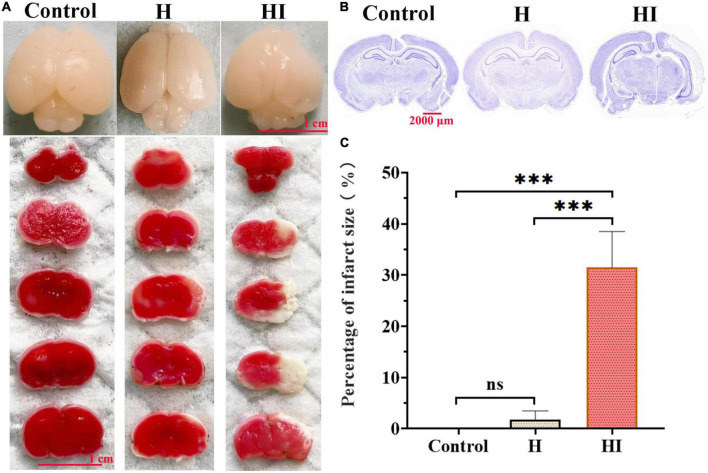
Morphological detection of HI-induced brain damage. **(A)** TTC staining detected the infarct size among control, H and HI groups. Scale bar = 1 cm. **(B)** Nissl staining images of whole brains in control, H and HI groups. Scale bar = 2,000 μm. **(C)** Quantification of brain infarct size among control, H and HI groups. Data were presented by Mean ± SD, ***p < 0.001.

With the intent to verify the reliability of the results obtained by TCD scans, TTC staining and Nissl staining were employed following TCD at 7th day to observe the pathological changes. Macroscopic images of whole brains demonstrated obvious atrophy and liquefaction in HI rats relative to that of H and control groups (Figure 5A). The results of Nissl staining showed that the nervous tissue of the HI group was significantly damaged compared with that of the H and control groups (Figure 5B). The stained brain tissues in the HI group exhibited much larger infarcted area than that in H and control groups indicated by higher percentage of infarct size in HI group (Figures 5A, C, *p* < 0.001). Moreover, correlation analysis revealed that the blood flow velocity of ACA and ICA-R was negatively correlated with the infarct size, with statistical significance in ACA (Supplementary Figure 1, *R*^2^ = 0.8591, *p* = 0.0235), and the blood flow velocity of ICA-R was positively correlated with the Zea-longa score in HI rats (Supplementary Figure 2, *R*^2^ = 0.8067, *p* = 0.0384).

## Discussion

In this study, TCD was used to detect cerebral vasculature and cerebrovascular flow of neonatal SD rats subjected to HI injury. The altered direction and acceleration of cerebral blood flow in ICA-L, MCA, and BA along with reduction of ICA-R flow were observed in neonatal HI rats under TCD. Morphological detection validated cerebral infarct and damage induced in rats, which was auxiliary to the TCD outcomes. The present study demonstrates that TCD is effectively applied in monitoring the dynamic cerebral blood flows of animals in a noninvasive way, which has an important guiding role in establishing NHIE models. TCD detection can properly treat and prevent cerebrovascular diseases in the future (Vagli et al., 2020). This study can provide better guidance if it can be combined with the analysis of clinical data.

Neonatal hypoxic-ischemic encephalopathy brings about severe neurological dysfunctions including cerebral palsy, mental retardation, cognitive impairment, and epilepsy, etc (Pin et al., 2009; Kurinczuk et al., 2010; Kyu et al., 2016; Xiao et al., 2020). Maximal medical support and hypothermia have been regarded as the current standard measurement of caring for moderate to severe NHIE, which reduce mortality and disability and improve neurocognitive function in HIE survivors (Shankaran et al., 2005; Gotchac et al., 2021). The capacity of inducing a controlled and consistent insult in experimental animals allows investigating the cellular and molecular mechanisms of post-injury brain damage and testing novel potential therapeutic drugs. In Rice et al. (1981) developed the Rice-Vannucci HIE model suffering permanent ligation of TCA-R and exposure to hypoxic environment at 37°C for 2.5 h, which would cause moderate or severe brain injury over 50 h. To date, Rice-Vannucci HIE model remains the most successful HIE model since it covers the defects of other modeling approaches and improves the efficiency of HI modeling, which is widely used in the preclinical investigations on the mechanisms and treatment of cerebrovascular diseases (Levine, 1960; Rice et al., 1981; Huang et al., 2017). The modeling effect of this model depends on the ligation of the right common carotid artery and the establishment of the hypoxic environment. The successful modeling mimicking HI injury is supported by a series of morphological detection (TTC staining, Nissl staining, HE staining, etc.) and behavioral evaluation including Zea-Longa score, Neurological Severity Score (NSS), Morris Water Maze test, Y maze test, Rotarod test, negative geotaxis test, etc. (Arteni et al., 2003). However, the ligation of the right common carotid artery is particularly important and easily influenced by the operation of the experimenters. So far, TTC staining is extensively used to distinguish the success of cerebrovascular diseases modeling (Zhang et al., 2019). Nissl staining and HE staining are also commonly used for observing the histological and cellular pathologies. However, these pathological changes are allowed to investigators based on the premise that all animal models used must be sacrificed. In this case, direct observation and trace of crucial indices are absent in living animals. Moreover, if tissues sections are poorly prepared and stained, the morphological identification and diagnosis become quite difficult. There is also a great possibility of inappropriate operation which results in modeling failure as well as even misleading experimental outcomes. In this study, TCD was applied to observe the cerebral vascular changes of NHIE rats. Our results revealed that TCD can accurately determine the intracranial vascular thickness and blood flow in neonatal SD rats, which can be used as an important basis for determining the success of NHIE modeling.

Cerebral blood flow is a useful biomarker in NHIE, and the assessment of cerebral blood flow in newborns with HIE can help detect abnormalities in brain perfusion to guide therapy and prognosticate patient outcomes (Tierradentro-García et al., 2021). Insults such as severe asphyxia, hypoxia and cerebral ischemia could accelerate the blood flow when the cerebral auto-regulation was impaired. The accelerated blood flow might be necessary to supply sufficient oxygen and glucose to the white matter of the peripheral regions and may represent autoregulation of cerebral vessels (Okuma et al., 2021). Alterations of cerebral blood flow in NHIE have been evaluated by distinctive neuroimaging techniques including contrast-enhanced ultrasound, arterial spin labeling MRI, 18F-FDG positron emission tomography, etc (Fantini et al., 2016). The complicated procedures and long-time consumption of them stand in the way of their wide application on laboratory animals. As a well-established research tool, TCD has been widely used to diagnose newborns HIE (Luo et al., 2022). A contemporary prospective cohort focusing on mild HIE obtained TCD measures shortly after birth and were classified in the first day after birth (Natique et al., 2021). Using TCD, the blood flow velocities and resistance index can be obtained to monitor the cerebral hemodynamics as well as auto-regulation (Krishna et al., 2018; Lin et al., 2018; Bonow et al., 2019; Natique et al., 2021). Actually, in humans, the MCA and its branches are the most often affected by stroke cerebral vessels up to 70% of all cerebral infarctions (Bogousslavsky et al., 1988). In a rat MCAO model, the insult completely blocks direct blood flow from ICA to MCA and the reverse blood flow from ACA (Gubskiy et al., 2018). Similarly, our TCD results directly displayed that the blood flow from ICA to MCA was blocked in HI rats, blood flow in the MCA and ICA-R was reversed due to reduction of ICA blood flow. Besides, blood back-flow was even found in the BA and ACA in the HI rats. Importantly, as times went by, the inverse flow in the BA was augmented. Meanwhile, the results showed that the flow rate of ICA-R was significantly lower in the HI rats than that in the H group, but the blood flow rate of BA and ICA-L was significantly higher in the HI group than that in the H group. This can be explained by the inability of the ICA-R to supply blood intracranially due to ligation. The compensatory increase in flow rate of the ICA-L and BA to satisfy the whole brain and the left blood supply compensates to the right side due to the presence of a cerebral arterial circle, which is opposite to the direction of normal common carotid artery blood flow. These experimental results indicate that the ligation of ICA-R is a decisive factor for cerebral vasculature and cerebrovascular flow. Correlation analysis further indicated blood flow in ICA-R is negatively correlated with infarct size of HI rats, but positively correlated with Zea-longa scores. Therefore, the direction and speed of cerebrovascular flow can be used to determine whether the ligation of ICA-R is complete and whether the NHIE modeling is successful, exhibiting important guiding significance for NHIE modeling.

Cerebrovascular disorders induce inadequate blood flow into corresponding brain regions and result in insufficient delivery of oxygen and glucose, which further induces tissues damage and loss. The main trunks of ACA head dorsally to the pial surface along the midline area, and the branches are mainly located in the hypothalamus, striatum, frontal lobe, olfactory bulb, and dorsal part of the cerebral cortex (Galasso et al., 2000). MCA is the middle branch of the ICA, and its branches inside the brain are mainly located in the hypothalamus, striatum, and amygdala (Cotten et al., 2014). BA is located in the ventrum of the medulla and pons, it extending to the ventral part of the thalamus. Hence, we also evaluated the morphological changes of HI rats by TTC staining and Nissl staining, demonstrating that large brain areas were damaged and infarcted including striatum, hypothalamus, frontal lobe, olfactory bulb, and dorsal part of the cerebral cortex, etc. Infarct in these different brain regions brings about specific functional barriers in neurodevelopmental functions such as memory, learning, language, sensory, motor, or coordination (Wei et al., 2022), etc.

A previous study reported hypoxic rats exhibited the similar cerebral damage to adult HIE rats in the early stage (Hou et al., 2019). In this study, we also applied TCD on hypoxic rats to compare its efficacy in distinguishing HIE newborns and asphyxiated neonates. As some studies discovered that sole ligation of cerebral artery cannot induced satisfying cerebral infarction in animal models as a result of Willis circle contralateral compensation (Kadam et al., 2010; Lyu et al., 2021), the solely ischemic rats were not taken into our consideration in this study. In HIE newborns, the blood flows in the ICA-L and BA were much more elevated than those in hypoxic only rats. Outcomes of TTC staining and Nissl staining in this study also corroborated the HI-induced cerebral morphological changes, which authenticated the applicable efficacy of TCD in monitoring NHIE modeling instead of hypoxia modeling. TCD enables real-time and accurate detection of cerebral vascular and blood flow velocities of NHIE rats, and exhibits the non-invasive, portable, low cost, and radiation-free advantages compared with other imaging methods (Topcuoglu, 2012; LaRovere, 2015; Blanco and Abdo-Cuza, 2018). All these advances highlight its important role of real-time visualization of cerebral vasculature and blood flow rates for understanding the occurrence and progression of NHIE.

## Conclusion

The cerebrovascular flow indicating the severity of NHIE rats can be monitored by TCD in a real-time and non-invasive way. The findings in this study are promising for the conclusion that TCD exhibits great potentials to revealing the biological properties indicating severity of NHIE and determining the successful establishment of NHIE model.

## Data availability statement

The original contributions presented in this study are included in the article/Supplementary material, further inquiries can be directed to the corresponding authors.

## Ethics statement

This animal study was reviewed and approved by the Ethics Committee of Kunming Medical University (approval number: kmmu20220748).

## Author contributions

JL, CF, and KZ performed the experiment and collected the data. CF and QW completed the manuscript. LX and TW gave instructions on the whole process. CF and YL provided the modification. LC participated in the article submission. RM and HZ participated in the supplementary experiment. All authors have read and approved the final submitted manuscript.
