# Supplementary material for: Transcranial Doppler Ultrasonography detection on cerebrovascular flow for evaluating neonatal hypoxic-ischemic encephalopathy modeling
Source: Front Neurosci. 2023 May 12;17:962001. doi: 10.3389/fnins.2023.962001 (PMC10213400; doi:10.3389/fnins.2023.962001)
Supplement: Supplementary file 1 [file Data_Sheet_1.pdf]

Supplementary figures

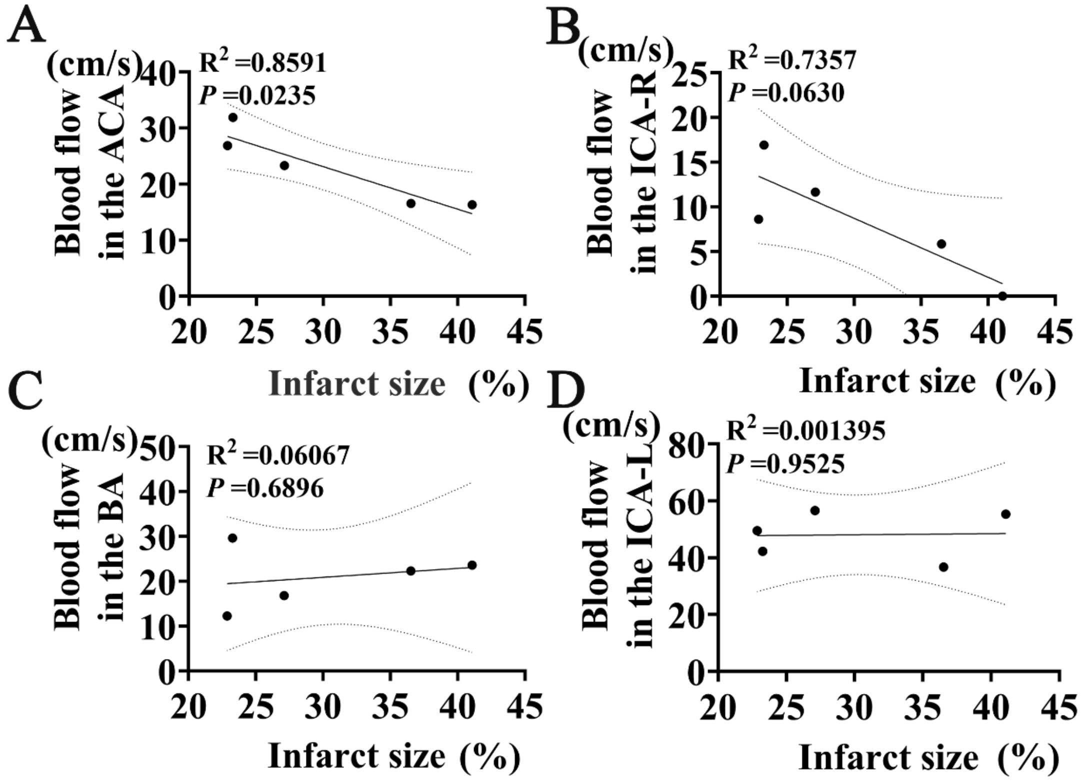

Supplementary Figure 1: Correlation analysis between vascular blood flow velocity measurement and infarct size 7 days after HI
Correlation analysis between blood flow velocity and infarct size of (A) ACA, (B) ICA-R, (C) BA and (D) ICA-L on the 7th day of HI. ACA, anterior cerebral artery; BA, basilar artery; ICA-L, left internal carotid artery; ICA-R, left internal carotid artery; HI, hypoxia and ischemia. N = 5.

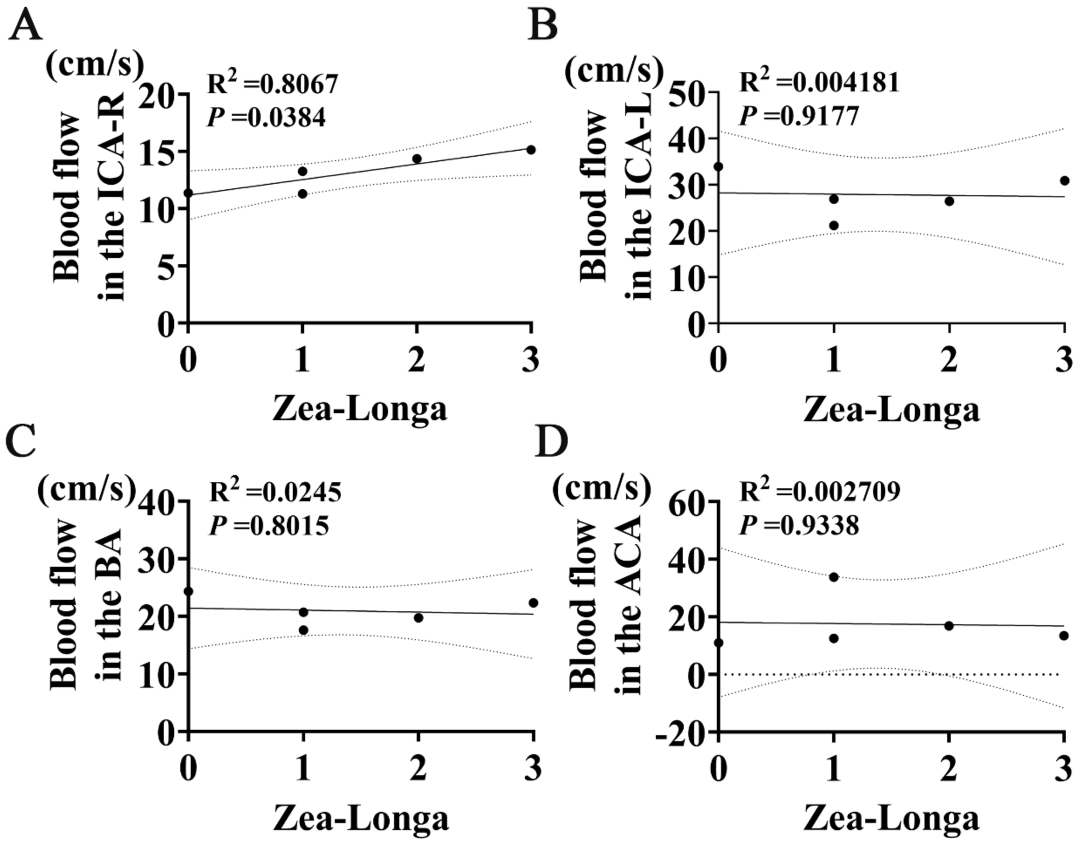

Supplementary Figure 2: Correlation analysis between vascular blood flow velocity measurement and Zea-Longa score at 2 days after HI. Correlation analysis between blood flow velocity and Zea-Longa score of(A) ICA-R, (B) ICA-L, (C) BA and (D) ACA at 2 days after HI. ACA, anterior cerebral artery; BA, basilar artery; ICA-L, left internal carotid artery; ICA-R, left internal carotid artery; HI, hypoxia and ischemia. N = 5.
